# The Role of C-Reactive Protein in Kidney, Bladder, and Prostate Cancers

**DOI:** 10.3389/fimmu.2021.721989

**Published:** 2021-08-27

**Authors:** Daniel O’Brian, Megan Prunty, Alexander Hill, Jonathan Shoag

**Affiliations:** ^1^Northwestern University, Feinberg School of Medicine, Chicago, IL, United States; ^2^Urology Institute, University Hospitals Cleveland Medical Center, Cleveland, OH, United States; ^3^Case Western Reserve University School of Medicine, Cleveland, OH, United States

**Keywords:** CRP - C-reactive protein, renal cell cancer (RCC), bladder cancer, urothelial carcinoma, prostate adenocarcinoma

## Abstract

C-Reactive Protein (CRP) is associated with diverse outcomes in patients with, or suspected to have, genitourinary malignancies. CRP levels have been shown to be associated with the probability of a prostate cancer diagnosis in patients with elevated PSA, the probability of biochemical recurrence following definitive treatment for localized prostate cancer, and decreased overall survival for patients with advanced disease. In patients with bladder and kidney cancers, CRP levels have been associated with disease progression, stage, and cancer-specific survival. Despite the abundance of correlative studies, the relationship between CRP levels and genitourinary cancer pathogenesis is not clearly understood. Here, we review the evidence for CRP as a biomarker in genitourinary (GU) cancers, with specific focus on potential clinical applications.

## Introduction

C-reactive protein (CRP) is an acute phase reactant that is widely considered to be a marker of both acute and chronic systemic inflammation. CRP secretion by hepatocytes is controlled by pro-inflammatory cytokines, namely interleukin-6 (IL-6) with additional influence from interleukin-1 (IL-1) and tumor necrosis factor (TNF) (1, 2). Because CRP’s half-life is long (19 hours) it is a stable marker for inflammation, unlike many cytokines whose half-lives can be as short as several minutes (1). Elevations in IL-6 and CRP correlate with cancer related anemia, and it has been postulated that states of persistent inflammation may induce a hypermetabolic state leading to malnutrition and cachexia (3).

CRP is a sensitive but nonspecific biomarker for inflammation. High baseline CRP in healthy patients carries an increased risk of future cancer development, although genitourinary cancers specifically have not been shown to be significantly correlated with CRP (4). Two main hypotheses exist regarding CRP elevation in cancer pathogenesis; one hypothesis suggests that CRP elevation occurs secondary to the inflammation of tumor growth, whereas the second suggests that CRP elevation is caused by the tumor itself (5).

## CRP in Renal Cell Carcinoma

The value of CRP has been supported by numerous studies for both evaluation and treatment of renal cell carcinomas (RCC). RCC directly secretes IL-6, which in turn is a direct stimulator of CRP secretion by hepatocytes (6). In a study of 143 patients (122 with RCC, 21 control), prominent pro-inflammatory cytokines (IL-6, TNFa, and IL-1B) were all strongly correlated with CRP levels. The tumor microenvironment may also play a role in cancer pathogenesis and be reflected in CRP measurements. In a prospective study of 111 patients undergoing either partial or radical nephrectomy for RCC in whom CRP was measured preoperatively, tumor infiltration of CD8 T-cells, M2 macrophages, and regulatory T cells on immunohistochemical staining were associated with elevated CRP, which, in turn, correlated with worse cancer specific survival (CSS) (**Table 1**). The authors hypothesize that regulatory T cells and M2 macrophages create an immunosuppressive microenvironment, which is reflected in CRP measurements (26).

**Table 1:** The association of elevated CRP levels with clinical response for kidney, bladder, and prostate cancer.

	Staging	Overall Survival	Disease Free Survival	Response to therapy
Kidney	Larger tumor higher grade and stage (4, 9–11)	Decreased (7, 8)	Decreased (7, 8)	Worse response to ICI, TKI, and cytokine therapies; CRP “flare” predicts tumor shrinkage (12, 13)
Bladder – NMIBC	Higher tumor stage (14)	–	–	Persistent CRP elevation is associated with disease progression after BCG (14)
Bladder – MIBC	Tumor stage, lymph node metastases (5, 16–18)	Decreased (5, 17, 19)	Not associated (15)	Failure of CRP to normalize with GC or MVAC predicts shorter OS (20, 21)
Prostate	Associated with Gleason Score but not tumor stage (22, 23)	Decreased (24)	Decreased DFS; Shorter biochemical failure-free survival (22, 24)	For CRPC on docetaxel, higher CRP is associated with shorter OS (25)

BCG, Bacillus Calmette-Guérin Immunotherapy; CRPC, Castrate-resistant Prostate Cancer; GC, Gemcitabine and Cisplatin; ICI, Immune Checkpoint Inhibitors; MIBC, Muscle Invasive Bladder Cancer; MVAC, Methotrexate, Vinblastine, Doxorubicin, and Cisplatin; NMIBC, Non-muscle Invasive Bladder Cancer; TKI, Tyrosine Kinase Inhibitors.

CRP elevation is an independent predictor for survival, as well as tumor recurrence (7, 8). High CRP is also associated with numerous poor prognostic indicators including larger tumor size, higher grade and stage, lymphatic involvement, microvascular invasion, and aggressive histopathological findings such as spindle morphology and sarcomatoid morphology (4, 9–11). These findings have been consistent between both localized and metastatic renal cell carcinomas (mRCC), and across numerous subtypes including clear cell, chromophobe, and papillary RCCs (27–30).

CRP alone does not outperform current prognostic scores such as TNM classification and the Karnofsky Index (31, 32). However, CRP has been shown to improve prognostic accuracy when incorporated into existing models such as the UCLA Integrated Staging System (UISS) (33). When CRP was incorporated, the predictive accuracy of the UISS for predicting cancer specific mortality was increased by 3.7%. Separately, a retrospective analysis suggested that CRP elevation was predictive of poor outcomes when incorporated into the International Metastatic Renal-Cell Carcinoma Database Consortium (IMDC) model for patients receiving axitinib. Additionally, several novel models incorporating CRP have been developed which show superiority to existing models (19, 34, 35). Of these, recently developed models assessing albumin, CRP, and lactate dehydrogenase (ACL model) or albumin, CRP, and neutrophil-lymphocyte-ratio (ACN model) were both found to have superior predictive value over both the IMDC and Memorial Sloan Kettering Cancer Center models.

For informing therapy, elevated pretreatment CRP is an independent predictor for poor survival in patients undergoing treatment with nephrectomy, tyrosine kinase inhibitors (TKIs), PD-L1 antagonists, IL-2, and interferon alpha. However, greater value in predicting treatment response is gained from assessing the early changes in serum CRP after initiation of treatment. Stratification of patients into groups based on whether CRP remained high, decreased with treatment, or was already low before treatment has been shown to have excellent predictive value for estimating survival and recurrence. A 2017 prospective study by Teishima et al. found 2-year overall survival rates in mRCC treated with tyrosine kinase inhibitors to be 75.5% for low initial CRP, 62.5% when a high initial CRP decreases with treatment, and 7.4% when CRP remains elevated (12). Similarly, in patients with mRCC treated with nivolumab, CRP kinetics predict greater tumor shrinkage and progression free survival (PFS), with the greatest response seen in the subset of people who experience a “CRP-flare” (initial CRP doubling with decline to below baseline level within three months) after administration of nivolumab (13) CRP kinetics have been especially well validated in response to nivolumab and sunitinib, but have also been directly validated in axitinib, IL-2, sorafenib, and surgical approaches.

It is important to note that a 2014 retrospective study by Tsuchiya et al. which focused on sunitinib response found that high CRP was correlated with superior tumor size reduction in metastases only in tumors that were <20mm before treatment. In larger tumors, CRP level was not found to have any effect on mean size reduction (36). These findings could suggest that the utility of CRP becomes more limited as disease severity increases, although further research is needed.

## CRP in Bladder Cancer

The value of CRP as a prognostic indicator for patients with bladder cancer has been assessed in multiple clinical states.

CRP has prognostic significance for non-muscle invasive bladder cancer (NMIBC). In one multi-institutional prospective study, 1117 patients underwent CRP measurement prior to transurethral resection of bladder tumor (TURBT). In this setting, elevated preoperative CRP is associated with higher tumor stage, but was not independently correlated with disease recurrence (14). For the subset of patients in this study with NMIBC who underwent adjuvant BCG, CRP was found to be associated with subsequent disease progression (14). When added to the previously validated Club Urológico Español de Tratamiento Oncológico (CUETO) Scoring Model for recurrence and progression of NMIBC treated with BCG, the CUETO model was significantly more accurate for predicting disease progression (improved accuracy by +1.2%), but not more accurate for predicting disease recurrence, with the addition of CRP as a variable (14, 37). One hypothesis for these findings is that CRP is worse at predicting disease recurrence is that disease recurrence is limited to the mucosal layer of the bladder and may have less systemic inflammatory effects, whereas disease progression to muscle-invasive bladder cancer (MIBC) may have more systemic effects.

In the setting of MIBC, CRP has been used in outcome measures in the peri-cystectomy period. Elevated CRP prior to cystectomy predicts primary tumor stage, positive resection margins, and lymph node metastases (5, 16–18). Combining CRP with albumin [CRP-albumin ratio (CAR)] as a measure of preoperative nutritional status, can also predict survival outcomes after radical cystectomy (38). Pre-operative CRP alone is also predictive of CSS in MIBC (5, 18). When comparing patients with above and below average CRP levels, patients with above average CRP levels have a median CSS between 51 and 60 months shorter than those with average CRP (5, 17). Pre-cystectomy CRP is also predictive of OS (5, 17, 18). In a study of 240 patients undergoing radical cystectomy for bladder cancer, a new outcome prediction model was validated, called the TNR-C score. The TNR-C includes CRP, pathologic T score, lymph node density, resection margins, age, and tumor size; as TNR-C score increases, both CSS and OS decrease (5, 16). Although the majority of studies report CRP as a predictor of survival, Gondo et al. report that CRP failed as an independent parameter of disease specific survival (15).

There are specific limitations in the predictive value of CRP in the context of MIBC. For example, there is no correlation between CRP levels and previous number of transurethral resections of bladder tumors (TURBT), time between previous TURBT and radical cystectomy (RC), and previous BCG therapy. There is also no significant difference in CRP levels between men and women with MIBC, though survival differences between men and women are widely reported (5, 16, 39).

For patients with advanced and inoperable bladder cancer, primary treatment comes in the form of chemotherapy and radiation. In a prospective study of 67 patients with inoperable bladder cancer, CRP was measured one day prior to initiation of chemotherapy (gemcitabine and cisplatin); elevated CRP was associated with both shorter PFS and shorter OS (20). Further, in a prospective study of 108 patients with MIBC undergoing primary treatment with external beam radiation and cisplatin chemotherapy, failure of CRP levels to normalize after chemoradiation was associated with poor survival. Patients with normalized CRP after treatment had a 5-year CSS of 77% and those who remained elevated had a 5-year CSS of 50% (40). At time of treatment with chemotherapy for advanced urothelial carcinoma, stratifying patients as non-elevated CRP (57.5% of patients), elevated to normalized CRP (30% of patients), and non-normalized CRP (12.5% of patients) groups held significant prognostic value (21). Patients with non-elevated CRP had significantly longer overall survival than patients with normalized CRP and non-normalized CRP. Similarly, a study of 80 patients with advanced, inoperable bladder cancer undergoing second line MVAC chemotherapy concurred that patients whose CRP normalized after chemotherapy also had improved overall survival as compared to the non-normalized group. Their conclusion suggests that CRP kinetics are associated with prognosis and survival (21). These studies all conclude that, since CRP measurement is simple and inexpensive, it can serve as a nonspecific marker of response for patients with MIBC being treated with chemoradiation.

In looking towards the future of CRP as a tool for risk stratification of bladder cancer, it is important to understand CRPs role when included in other scoring systems. The Glasgow Prognostic Score (GPS) combines CRP and albumin as a measure of inflammation. GPS was first evaluated in lung cancer research and has since been applied in GU oncology, where it serves as an independent predictor of poor prognosis (41). The RLC Score (R status, lymphovascular invasion, C-reactive protein) combines age, comorbidities, pre-/postoperative serum levels of CRP, leukocytes, hemoglobin, creatinine, urinary diversion, tumor grading, staging, lymph node status, lymph node density (LND), lymphovascular invasion (LVI), metastases, and resection margin status (42). The RLC score identifies patients at a higher risk of overall mortality after radical cystectomy. The TNR-C score combines T-stage, lymph node density, resection margin status, and CRP into a risk stratification system for cancer-specific outcomes after radical cystectomy; it identifies preoperative CRP as an independent risk factor for cancer specific survival (CSS) and inclusion of CRP increases the predictive accuracy and c-index significantly (5, 17). In the setting of locally advanced and metastatic bladder cancer, both 6 and 12 month survival nomograms improved with the addition of CRP to traditional markers (age, ECOG, PS, Hb, LDH, visceral metastatic disease, and lymph node metastases) (43). For bladder cancer, the data suggest CRP is a useful biomarker, which can provide additional prognostic information when combined with existing metrics.

## CRP in Prostate Cancer

There is an increasing body of evidence for the role of inflammation in the pathogenesis of prostate cancer (PCa). Several proposed mechanisms exist for the role of inflammation in PCa, such as cellular and genomic damage, cellular turnover, promotion of an environment that promotes cellular replication, angiogenesis, and tissue repair (44, 45). In a population-based, prospective study of 7,270 men followed for a mean of 11.8 years with serial inflammatory marker measurements, a CRP rise ≥ 1.0 mg/l between two measurements purported a 36% increase in subsequent prostate cancer diagnosis. However, other studies have failed to show an independent association between CRP and the future development of prostate cancer (46–49).

Men who meet screening criteria and have persistently elevated prostate-specific antigen (PSA) often undergo prostate biopsy to diagnose cancer. CRP has been used in efforts to risk stratify these patients and distinguish benign prostatic enlargement from PCa. In a retrospective review of 251 patients undergoing prostate biopsy for PSA > 4.0 ng/mL, there was a statistically significant difference in the natural log of CRP between BPH, ≤T2, and ≥T3 cancers (50). Two additional studies have found no difference between CRP levels in patients with benign hyperplasia and localized prostate cancer, but found statistically significant differences in CRP level between benign hyperplasia and patients with metastatic prostate cancer (48, 51).

Limited data exists regarding CRP in the setting of localized prostate cancer treated with radical prostatectomy (RP). In a retrospective, multi-institutional study of 7,426 patients who underwent RP, pre-prostatectomy CRP levels were associated with pathologic Gleason score, but not tumor stage (22, 23). Similarly, patients with elevated preoperative CRP were more likely to have extracapsular extension, seminal vesicle invasion, lymph node metastasis, positive surgical margins, and biochemical recurrence (23). Although elevated postoperative CRP was associated with adverse pathologic outcomes and higher rates of 5-year biochemical recurrence, the addition of CRP to established pre-operative modeling for BCR did not improve predictive modeling over the traditional characteristics alone (23, 52). As such, CRP likely has limited utility for outcome prediction after RP.

CRP has prognostic potential for patients with prostate cancer treated with radiation. Based on one retrospective, institutional study of 1,500 patients treated with radiation, CRP was associated with PSA only in a limited cohort of patients (those with Gleason scores 8, pretreatment PSA levels >20 ng/mL, and those categorized as high risk). In this cohort, higher CRP was associated with shorter biochemical failure‐free survival (22). In a similar retrospective, institutional study of 700 patients undergoing radiation for prostate cancer, a pre-treatment CRP ≥8.6 mg/L was associated with worse cancer-specific survival, overall survival, and clinical disease-free survival. The association between elevated CRP and worse survival was independent of other risk factors, including stage, grade, and PSA (24). Future research is needed on the topic but in patients with PCa treated with radiation, CRP may be a useful prognostic tool.

Among patients with metastatic prostate cancer, the relationship between PSA and survival is well established. In evaluating a cohort of 62 patients with metastatic prostate cancer on androgen deprivation therapy, CRP and PSA were evaluated simultaneously; higher levels of both CRP and PSA were independent predictors of poorer cancer-specific survival. The values of CRP and PSA were significantly correlated, further confirming their prognostic potential (53).

For patients with castration-resistant prostate cancer (CRPC) on chemotherapy, elevated CRP is an independent predictor of overall survival and progression-free survival (25, 54, 55). Among 80 consecutive patients with CRPC being treated with docetaxel, CRP <5 mg/L showed a 14 month longer median survival time than those with CRP >5 mg/L. These patients were further categorized into risk groups based on a combination of CRP and hemoglobin level, with overall survival curves significantly different among the groups (25). Similarly, among 115 patients with CRPC on docetaxel, CRP ≥ 8 mg/L was associated with higher risk of tumor progression and shorter overall survival (55). Finally, a secondary analysis of frozen plasma samples from the AIPC Study of Calcitriol ENhancing Taxotere (ASCENT) trial, a randomized controlled trial, CRP was studied as a continuous variable and found to be a significant predictor of overall survival. An elevated CRP was also associated with decreased probability of PSA decline (54). It is critical to note that other studies have shown better outcomes in predicting response of CRPC to chemotherapy using factors such as age, PSA, serum hemoglobin, and serum alkaline phosphatase (56, 57).

The modified Glasgow Prognostic Score (mGPS) combines albumin and CRP as a measure of inflammation. In the setting of CRPC, a higher mGPS is associated with shorter overall survival, giving it prognostic potential (45, 58). A higher mGPS has also been shown to have a higher risk of death within both 5 and 10 years of follow-up. These associations remain even after controlling for socioeconomic factors, Gleason score, PSA, and number of in-patient days in 10 years preceding prostate cancer diagnosis (59). With further study, mGPS could prove to be useful as a prognostic indicator in patients with CRPC that undergo chemotherapy for their cancer.

Two meta-analyses, have found that elevated pretreatment CRP is associated with worse overall survival, cancer-specific survival, progression-free survival, and biochemical recurrence-free survival (55, 60). Although there were some independent associations between CRP and PCa outcomes, whether CRP meaningfully contributes to prognosis in the setting the multiple other prognostic factors used in PCa is uncertain.

## Discussion

For renal cell carcinoma, postoperative CRP levels and CRP kinetics hold the most predictive value. For bladder cancer, CRP levels are associated with disease progression in NMIBC and with stage and survival for MIBC. For prostate cancer, the role of CRP is fairly limited with prognostic utility observed in some, but not all settings. Currently, it is not our practice to assess CRP in patients undergoing GU cancer treatment, as to our knowledge it does not seem to add to existing prognostic models in a manner that would be meaningful for patients or providers.

## Author Contributions

DO’B, MP, AH, and JS all contributed to writing and proofreading manuscript. All authors contributed to the article and approved the submitted version.

## Conflict of Interest

The authors declare that the research was conducted in the absence of any commercial or financial relationships that could be construed as a potential conflict of interest.

## Publisher’s Note

All claims expressed in this article are solely those of the authors and do not necessarily represent those of their affiliated organizations, or those of the publisher, the editors and the reviewers. Any product that may be evaluated in this article, or claim that may be made by its manufacturer, is not guaranteed or endorsed by the publisher.
